# Drug-coated balloon in the treatment of coronary left main true bifurcation lesion: A patient-level propensity-matched analysis

**DOI:** 10.3389/fcvm.2022.1028007

**Published:** 2022-11-03

**Authors:** Liang Pan, Wenjie Lu, Zhanying Han, Sancong Pan, Xi Wang, Yingguang Shan, Meng Peng, Xiaofei Qin, Guoju Sun, Peisheng Zhang, Jianzeng Dong, Chunguang Qiu

**Affiliations:** ^1^Department of Cardiovascular Medicine, The First Affiliated Hospital of Zhengzhou University, Zhengzhou, China; ^2^Department of Cardiology, Jincheng People’s Hospital, Jincheng, China; ^3^Department of Cardiology, The Fifth Affiliated Hospital of Zhengzhou University, Zhengzhou, China

**Keywords:** drug-coated balloon, drug-eluting stent, left main, true bifurcation, *de novo*

## Abstract

**Aims:**

An increasing body of evidence suggests that drug-coated balloon (DCB) angioplasty represents a valuable option for revascularization in selected patients with coronary bifurcation disease. However, there remains a paucity of real-world observational evidence on the efficacy of DCB in left main (LM) true bifurcation lesion. We compared clinical and angiographic outcomes of hybrid [DCB + drug-eluting stent (DES)] versus DES-only strategy (provisional stenting or two-stent strategies) in *de novo* LM true bifurcated lesions.

**Methods:**

The primary endpoint was the 2-year composite rate of target lesion failure (TLF): cardiac death, target vessel myocardial infarction (TVMI), or clinically driven target lesion revascularization (CD-TLR). A routine 1-year angiographic follow-up was scheduled. Propensity-score matching was utilized to assemble a cohort of patients with similar baseline characteristics.

**Results:**

Among 1077 eligible patients, 199 who received DCB treatment and 398 who were assigned to DES therapy had similar propensity scores and were included in the analysis. TLF within 2 years occurred in 13 patients (7.56%) assigned to DCB group, and 52 patients (14.36%) assigned to DES group (odds ratio: 0.487; 95% confidence interval: 0.258–0.922; *P* = 0.025; Log-rank *P* = 0.024). Compared with the DES group, the DCB group resulted in a lower rate of CD-TLR (2.91% *vs.* 9.42%; *P* = 0.007). Cardiac death, TVMI, all-cause mortality, and stent thrombosis were comparable between both groups. Patients treated with DES-only were associated with a higher late lumen loss (0.42 ± 0.62 mm *vs*. 0.13 ± 0.42 mm, *P* < 0.001) compared with the DCB group at 1 year. In sensitivity analysis, the DCB group also presented a lower incidence of TLF, CD-TLR and stent thrombosis both compared to the two-stent strategy and compared to provisional stenting (*P*s < 0.05).

**Conclusion:**

The 2-year results of PCI utilizing DCB for LM true bifurcation lesions are superior to employing DES alone in terms of safety and effectiveness.

## Graphical Abstract

DCB, drug-coated balloon; LM, left main; PSM, propensity score matching; DES, drug-eluting stent; TLF, target lesion failure; CD-TLR, clinically driven target lesion revascularization. 
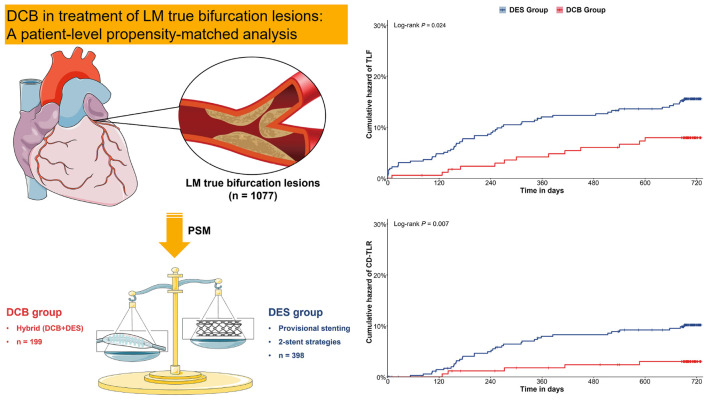

## Introduction

Left main (LM) bifurcation is consistently distinct from other bifurcations (1), and the side branch (SB) is often the circumflex (LCx), which is frequently angular and has a large reference diameter, making it challenging to treat. Oftentimes, LCx acute occlusion causes significant ischemia. It has been reported that the T-shaped bifurcation angle of the LM may also alter the implantation procedure, and a steeply angled LCx take-off may affect the prognosis following LM stenting. According to recent myocardial revascularization guidelines, patients with significant LM disease with a low or moderate SYNTAX score have a class I indication for percutaneous coronary intervention (PCI) (2). However, bifurcation stenting is frequently associated with greater rates of restenosis and thrombosis, especially in complex procedures (3). Provisional side branch intervention is preferred for most bifurcation lesions (3, 4). Nonetheless, provisional stenting may not always be appropriate for LM bifurcation lesions. Accordingly, further studies are essential to developing new technologies to reduce restenosis in bifurcation lesions.

Nowadays, drug-coated balloons (DCBs) can be used to deliver antiproliferative drugs without the need for implanting permanent prostheses. DCB has well-established efficacy in treating coronary in-stent restenosis (ISR) (5, 6) and *de novo* lesions (7–9). Notably, the past decade has witnessed significant inroads achieved in DCB technology, lesion preparation, and clinical experience for bifurcation PCI (10–12).

To the best of our knowledge, no study has hitherto explored the utilization of DCB in LM true bifurcation lesions. Our multicenter study retrospectively evaluates the effect of hybrid [DCB + drug-eluting stent (DES)] and DES-only techniques on 2-year outcomes in LM true bifurcation diseases.

## Materials and methods

### Study subjects

**FIGURE 1:**
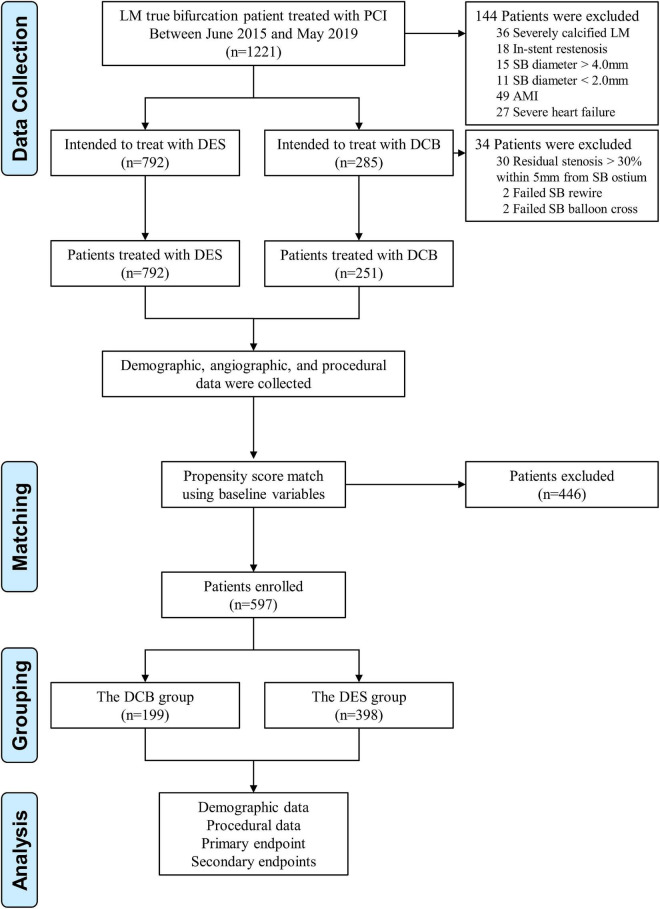
Study population. LM, left main; PCI, percutaneous coronary intervention; SB, side branch; AMI, acute myocardial infarction; DCB, drug-coated balloon; DES, drug-eluting stent.

Patients were enrolled consecutively from June 2015 to May 2019 at three Chinese centers (including the First Affiliated Hospital of Zhengzhou University, Jincheng People’s Hospital, and the Fifth Affiliated Hospital of Zhengzhou University). All patients submitted written informed permission, which was authorized by the Ethics Committee of the First Affiliated Hospital of Zhengzhou University. Patients that exhibited stable coronary disease or unstable angina pectoris and underwent PCI for *de novo* coronary lesions (diameter stenosis > 50%) at the LM bifurcation (Medina 1,0,1, 0,1,1 or 1,1,1) (13), with an SB diameter ≥ 2.0 mm, were included in the present study. Patients with the following criteria were excluded: (1) angiographic evidence of severely calcified LM lesions requiring atherectomy; (2) ISR; (3) Inherence to the procedural steps of optimization defined below as extracted from angiographic images and reports reviews; (4) acute myocardial infarction (MI); (5) cardiogenic shock or unstable hemodynamics (Figure 1).

Patients received aspirin (300 mg loading dose) or long-term aspirin medication prior to the intervention. A loading dosage of 600 mg of clopidogrel or 180 mg of ticagrelor was given. Dual antiplatelet treatment (DAPT) was provided for the duration recommended by guidelines (2, 14). Women of reproductive potential and those with a life expectancy less than one year, as well as patients with contraindications to DAPT, bivalirudin, heparin, paclitaxel, or –*limus*, were excluded from the study. A standard computerized case report form was used to collect the data.

### Study procedures

In the DES group, LM true bifurcation lesions were treated with any solution using DES alone, in accordance with the recommendations of the European Bifurcation Club (EBC) (1), including provisional SB interventional approach or any two-stent strategies.

**FIGURE 2:**
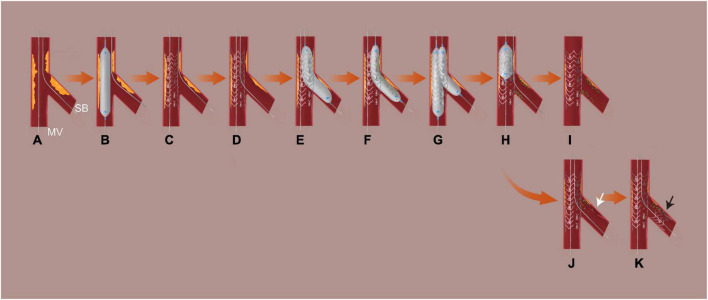
Procedural steps in DCB group. **(A)** Wiring both branches; **(B)** MV lesion preparation; **(C)** MV stent implantation; **(D)** rewire SB from the distal strut; **(E)** SB lesion preparation: plaque modification and expand the orifice of SB; **(F)** SB DCB angioplasty; **(G)** final kissing inflation; **(H)** POT if necessary; **(I)** final result I; **(J)** dissection (≥type C or flow-limited, white arrow); **(K)** bailout stent deployment (black arrow, the distance between the proximal end of the stent and the ostium of the vessel ≥ 3 mm) and final result II. MV, denotes main vessel; NC, non-compliant; SB, side branch; DCB, drug-coated balloon; POT, proximal optimizing technique. If there was a residual stenosis ≤ 30% only in proximal 5 mm regardless of other segment after SB lesion preparation, a DCB + DES strategy was initially selected. DCB angioplasty was done first, followed by stent deployment to ensure complete coverage of the dissection and severe elastic recoil segment (residual stenosis > 30%). The distance between the proximal end of the stent and the ostium of SB ≥ 3 mm.

In the DCB group, appropriate lesion preparation was emphasized prior to DCB treatment. Both branches were wired, and the main vessel (MV) was pre-dilated. Subsequently, the stent was implanted in the MV and fully expanded. Then, the SB was rewired from the distal strut mesh, and SB lesion preparation was conducted (plaque modification and expand the orifice of SB). Dilatation was required using a plain or non-compliant balloon with a balloon-to-vessel ratio of 0.8–1.0. In the absence of a major, flow-limiting dissection [<Type C according to the NHLBI classification (15)] and if residual stenosis was ≤ 30% based on at least two perpendicular angiographic views, DCB angioplasty was conducted. For residual stenosis ≤ 30% only in the proximal 5 mm following lesion preparation, irrelevant of other segments, a DCB + DES approach was initially selected in SB. To achieve full coverage of the dissection and severe elastic recoil segment, DCB angioplasty was performed first, followed by stent placement. The distance between the stent’s proximal end and the SB ostium should be more than 3 mm. Patients with residual stenosis > 30% within 5 mm of the SB ostium after SB lesion preparation were eliminated (switching to a 2-stent strategy or SB palliative DCB angioplasty). Finally, kissing balloon inflation and the proximal optimizing technique (POT) were conducted. In this investigation, the DCB used was coated with a matrix of paclitaxel and iopromide (SeQuent™ Please, B. Braun, Melsungen, Germany). To avoid a geographic mismatch, the DCB catheter was extended 4–5 mm into the MV and 2–3 mm beyond the pre-dilated area. Using a balloon-to-vessel ratio of 0.8–1.0, the DCB diameters were matched to the reference vessel diameters. At a pressure of > 7 bars, the suggested inflating time was at least 30 s. If the outcome of DCB treatment was unsatisfactory owing to significant residual stenosis or dissections, a new-generation DES was implanted (Figure 2).

Non-target lesions were first treated if present.

### Follow-up

For 2 years, clinical follow-up was done through office visits or telephone calls every 3 months. Following evaluating the main clinical endpoint, coronary angiography was planned for all patients 12 months after the index procedure, unless clinical indications warranted an earlier angiographic evaluation.

### Endpoint and definitions

Target lesion failure (TLF), composed of cardiac death, target vessel myocardial infarction (TVMI), or clinically driven target lesion revascularization (CD-TLR) after 2 years of follow-up, was the primary endpoint. When the cause of death was undetermined or unknown, cardiogenic causes were assumed. Periprocedural myocardial infarction (≤48 h) was defined as cardiac troponin values at least five times the 99th percentile upper reference limit (URL) of the assay plus either: (1) new ischemic ECG changes or development of new pathological Q waves; (2) imaging evidence of new loss of viable myocardium or new regional wall motion abnormality; or (3) angiographically documented graft or coronary artery occlusion or new severe stenos. Spontaneous myocardial infarction (after 48 h) was defined as a clinical symptom consistent with MI with cardiac troponin values > 1 × URL and new ST-segment elevation or depression or other abnormalities as described above (16). All MIs were deemed TVMIs unless it could be proven that they were caused by non-target vessels (17). Angina or ischemia due to the target lesion necessitating repeat PCI or coronary artery bypass graft (CABG) surgery because of restenosis or thrombosis of the target lesion, which encompassed the proximal and distal edge segments of both branches, was defined as CD-TLR. Secondary outcomes, including all-cause mortality, cardiac death, TVMI, CD-TLR, and stent thrombosis (ST), were also assessed. ST was defined in line with the definition provided by the Academic Research Consortium (17).

### Quantitative coronary angiographic assessment

For quantitative coronary angiographic (QCA) analysis, edge detection methods and a bifurcation algorithm (QAngio XA version 7.3, Medis Medical Imaging, Leiden, Netherlands) were used (18). The guiding catheter was employed as the reference for calibration. Measurements were performed at baseline, after the procedure and during follow-up angiography. The MV was determined based on the anatomical nature of the lesion. The QCA variables analyzed included: (a) lesion length; (b) reference vessel diameter (RVD); (c) minimal lumen diameter (MLD); (d) percent diameter stenosis; (e) acute luminal gain (MLD immediately after the procedure minus the MLD before the procedure); and (f) late lumen loss (LLL) (MLD immediately after the procedure minus the MLD at follow up).

### Statistical analysis

All findings were analyzed using R version 3.6.1 (The R Foundation, Vienna, Austria^1^) and EmpowerStats (R) (X&Y Solutions Inc., Boston, MA, USA^2^). Categorical data were reported as frequencies (percentages), whereas continuous variables were presented as means ± standard deviation. Using Fisher’s exact test for categorical variables and the Mann–Whitney *U* test (Wilcoxon rank sum test) for continuous variables, the DCB and DES groups were compared.

Due to disparities in baseline characteristics between eligible participants in the two groups of the observational trial, 1:2 propensity score matching (PSM) was utilized to select patients with similar baseline data. After evaluation of covariates associated clinically and/or statistically with the treatment group and removal of repeatedly defined or collinear variables, including baseline characteristics, risk factors, clinical conditions at admission and treatment during operation, 13 variables (including age, sex, diabetes mellitus, hypertension, hyperlipidemia, renal insufficiency, clinical presentation, family history of coronary artery disease, smoking history, prior PCI history, previous MI history, previous CABG history, and left ventricular ejection fraction) were included in the PSM model using greedy (nearest neighbor) matching without replacement and a caliper of 0.02 (Supplementary Figure 1). In both the whole population and the propensity-matched cohort, the primary and secondary outcomes (DCB *vs.* DES) were evaluated. Using a log-rank test, the outcomes were compared and shown as Kaplan–Meier (KM) curves.

According to age (<60 years, or ≥60 years), gender (male or female), diabetes status (yes or no), and SYNTAX score (0–22, 23–32, or >32), subgroup analyses were conducted. To ensure a baseline balance between the DCB and DES groups throughout subgroup analysis, only matched pairings within a subgroup were selected. For instance, for diabetic patients, only matched pairs of diabetic patients in the DCB and DES groups were included in the study. To evaluate heterogeneity of treatment impact across subgroups, stratified analyses were conducted.

Separate propensity-matched cohorts of patients who received PCI with DCB as opposed to those who had the 2-stent technique and those who underwent PCI with DCB as compared to those who underwent provisional stenting were also assessed for the 2-year primary and secondary outcomes.

For all reported analyses, a two-sided *P*-value < 0.05 was statistically significant.

## Results

### Baseline clinical, angiographic, and procedural characteristics

A total of 1,077 LM true bifurcation patients treated with PCI met our inclusion and exclusion criteria. Of these, 792 patients were intended to treat with DES, and the remaining 285 patients were intended to treat with DCB. Finally, 34 patients with residual stenosis > 30% within 5 mm from SB ostium (*n* = 30); failed SB rewiring (*n* = 2) and failed SB balloon cross (*n* = 2) were excluded in the DCB group (Figure 1). Baseline characteristics are shown in Table 1. After matching, 597 patients (199 in the DCB group and 398 in the DES group) were selected. Statistical differences between the groups in age, renal insufficiency, and left ventricular ejection fraction were reduced upon patient matching.

**TABLE 1:** Demographic characteristics before and after propensity-score matching*.

Variable	All patients			Propensity-matched sample		
		
	DCB group (*n* = 251)	DES group (*n* = 792)	*P*-value	DCB group (*n* = 199)	DES group (*n* = 398)	*P*-value
Age (years)	61.28 ± 11.14	63.91 ± 7.76	0.001	63.75 ± 8.13	64.48 ± 7.75	0.261
Sex (Male)	188 (74.90%)	586 (73.99%)	0.774	145 (72.86%)	299 (75.13%)	0.551
**Comorbidity**						
Diabetes mellitus	96 (38.25%)	299 (37.75%)	0.888	77 (38.69%)	165 (41.46%)	0.517
Hypertension	125 (49.80%)	399 (50.38%)	0.873	107 (53.77%)	205 (51.51%)	0.602
Hyperlipidemia	63 (25.10%)	241 (30.43%)	0.105	54 (27.14%)	119 (29.90%)	0.483
History of smoking	89 (35.46%)	295 (37.25%)	0.609	72 (36.18%)	138 (34.67%)	0.716
Renal insufficiency	9 (3.59%)	61 (7.70%)	0.023	7 (3.52%)	24 (6.03%)	0.192
**Clinical presentation**			0.433			0.478
Stable angina	91 (36.25%)	309 (39.02%)		75 (37.69%)	162 (40.70%)	
Unstable angina	160 (63.75%)	483 (60.98%)		124 (62.31%)	236 (59.30%)	
Previous MI history	15 (5.98%)	56 (7.07%)	0.549	11 (5.53%)	26 (6.53%)	0.631
Previous PCI history	42 (16.73%)	138 (17.42%)	0.801	31 (15.58%)	74 (18.59%)	0.362
Previous CABG history	4 (1.59%)	10 (1.26%)	0.691	3 (1.51%)	7 (1.76%)	0.822
Family history of CAD	52 (20.72%)	188 (23.74%)	0.322	43 (21.61%)	90 (22.61%)	0.781
LVEF	59.59 ± 5.78	58.27 ± 6.15	0.005	59.64 ± 5.21	59.38 ± 5.60	0.941

*Plus–minus values are means ± SD. DCB, drug-coated balloon; DES, drug-eluting stent; MI, myocardial infarction; PCI, percutaneous coronary intervention; CABG, coronary artery bypass grafting; CAD, coronary artery disease; LVEF, left ventricular ejection fraction; DES, drug-eluting stent.

Procedural and angiographic baseline features are shown in Tables 2, 3. There were 597 lesions after matching, of which 199 (33.33%) were treated with DCB (hybrid strategy) and 398 (66.67%) with DES-only strategy (DK crush 113; Culotte 90; T stent 18; kissing stent 4; and provisional stenting 173). The mean SYNTAX score of the matched cohort was 32.23 (±7.08), with multivessel disease present in 87.60% of cases. The distal LM bifurcation lesion was classified as Medina 1,1,1; 0,1,1; and 1,0,1 in 80.90%, 10.22%, and 8.88% of cases, respectively and 18.93% were trifurcations lesions. The lesion characteristics of the two groups were similar before and after matching, including RVD, stenosis (%), and lesion length (*P*s > 0.05).

**TABLE 2:** Lesion characteristics before and after propensity-score matching*.

Variable	All patients			Propensity-matched sample		
		
	DCB group (*n* = 251)	DES group (*n* = 792)	*P*-value	DCB group (*n* = 199)	DES group (*n* = 398)	*P*-value
Classifications			0.120			0.376
Medina 0,1,1	19 (7.57%)	87 (10.98%)		16 (8.04%)	45 (11.31%)	
Medina 1,1,1	214 (85.26%)	629 (79.42%)		167 (83.92%)	316 (79.40%)	
Medina 1,0,1	18 (7.17%)	76 (9.60%)		16 (8.04%)	37 (9.30%)	
Trifurcation	52 (20.72%)	141 (17.80%)	0.300	44 (22.11%)	69 (17.34%)	0.160
**MV**						
Total occlusion	9 (3.59%)	29 (3.66%)	0.955	7 (3.52%)	19 (4.77%)	0.478
Intracoronary thrombus	2 (0.80%)	2 (0.25%)	0.246	2 (1.01%)	2 (0.50%)	0.604
Calcified lesions	76 (30.28%)	214 (27.02%)	0.315	60 (30.15%)	112 (28.14%)	0.609
Reference diameter (mm)	3.30 ± 0.22	3.32 ± 0.23	0.246	3.29 ± 0.22	3.31 ± 0.22	0.254
MLD (mm)	0.93 ± 0.34	0.96 ± 0.35	0.148	0.91 ± 0.34	0.95 ± 0.37	0.186
Diameter stenosis (QCA)	0.72 ± 0.10	0.71 ± 0.10	0.210	0.72 ± 0.10	0.71 ± 0.11	0.240
Area stenosis (QCA)	0.91 ± 0.05	0.91 ± 0.06	0.210	0.91 ± 0.05	0.91 ± 0.06	0.240
Lesion length (mm)	31.73 ± 14.66	30.07 ± 12.54	0.500	32.63 ± 15.05	30.38 ± 13.05	0.189
**SB**						
Total occlusion	8 (3.19%)	25 (3.16%)	0.981	6 (3.02%)	10 (2.51%)	0.720
Intracoronary thrombus	2 (0.80%)	7 (0.88%)	1.000	1 (0.50%)	4 (1.01%)	0.669
Calcified lesions	23 (9.16%)	82 (10.35%)	0.585	20 (10.05%)	45 (11.31%)	0.642
Severe tortuous	63 (25.10%)	226 (28.54%)	0.289	53 (26.63%)	108 (27.14%)	0.896
Reference diameter (mm)	3.01 ± 0.49	2.97 ± 0.32	0.264	3.00 ± 0.49	2.98 ± 0.32	0.584
MLD (mm)	0.97 ± 0.40	0.99 ± 0.36	0.786	0.95 ± 0.40	1.02 ± 0.35	0.069
Diameter stenosis (QCA)	0.67 ± 0.12	0.67 ± 0.12	0.857	0.67 ± 0.12	0.66 ± 0.11	0.225
Area stenosis (QCA)	0.88 ± 0.07	0.88 ± 0.07	0.833	0.88 ± 0.07	0.87 ± 0.07	0.317
Lesion length (mm)	15.25 ± 6.96	15.02 ± 6.94	0.636	15.51 ± 7.07	15.11 ± 6.80	0.490
Bifurcation angle (Degrees)	78.89 ± 23.16	78.78 ± 23.11	0.963	78.47 ± 22.82	76.84 ± 23.02	0.427
Multivessel disease	223 (88.84%)	701 (88.51%)	0.885	175 (87.94%)	348 (87.44%)	0.861
SYNTAX score	32.51 ± 7.13	31.93 ± 6.88	0.146	32.47 ± 7.24	32.11 ± 7.01	0.440
0–22	28 (11.16%)	91 (11.49%)	0.884	24 (12.06%)	50 (12.56%)	0.978
23–32	87 (34.66%)	286 (36.11%)		67 (33.67%)	135 (33.92%)	
>32	136 (54.18%)	415 (52.40%)		108 (54.27%)	213 (53.52%)	

*Plus–minus values are means ± SD. DCB, drug-coated balloon; DES, drug-eluting stent; MV, main vessel; SB, side branch; MLD, minimal lumen diameter; QCA, quantitative coronary angiographic; SYNTAX, synergy between percutaneous coronary intervention with taxus and cardiac surgery.

**TABLE 3:** Procedural characteristics before and after propensity-score matching*.

Variable	All patients			Propensity-matched sample		
		
	DCB group (*n* = 251)	DES group (*n* = 792)	*P*-value	DCB group (*n* = 199)	DES group (*n* = 398)	*P*-value
Transradial approach	239 (95.22%)	711 (89.77%)	0.008	191 (95.98%)	361 (90.70%)	0.021
6F guiding catheter used	174 (69.32%)	425 (53.66%)	<0.001	133 (66.83%)	223 (56.03%)	0.011
MV lesion preparation (pre-dilation)	251 (100.00%)	792 (100.00%)	-	199 (100.00%)	398 (100.00%)	-
MV stent						
Diameter (mm)	3.19 ± 0.39	3.19 ± 0.40	0.992	3.16 ± 0.39	3.17 ± 0.39	0.959
Total length (mm)	37.84 ± 14.60	36.20 ± 12.70	0.458	38.69 ± 15.05	36.49 ± 13.19	0.248
Covered ostial LM	148 (58.96%)	478 (60.35%)	0.695	116 (58.29%)	234 (58.79%)	0.906
SB lesion preparation						
Semi-compliant balloon	251 (100.00%)	792 (100.00%)	-	199 (100.00%)	398 (100.00%)	-
Non-compliant balloon	205 (81.67%)	316 (39.90%)	<0.001	164 (82.41%)	161 (40.45%)	<0.001
Maximum pre-dilation balloon diameter (mm)	2.86 ± 0.52	2.30 ± 0.33	<0.001	2.84 ± 0.51	2.31 ± 0.34	<0.001
Maximum pre-dilation balloon diameter/RD ratio	0.93 ± 0.08	0.78 ± 0.07	<0.001	0.93 ± 0.08	0.77 ± 0.07	<0.001
SB stent (2-stent strategies)						
Number of DESs used (per lesion)	–	1.12 ± 0.329	–	–	1.13 ± 0.34	–
Diameter (mm)	–	2.82 ± 0.31	–	–	2.81 ± 0.30	–
Total length (mm)	–	22.07 ± 7.46	–	–	22.37 ± 7.54	–
DCB/DES use (hybrid strategy)						
Number of DCBs used (per lesion)	1.01 ± 0.11	–		1.02 ± 0.12	–	
DCB diameter (mm)	2.89 ± 0.39	–		2.88 ± 0.39	–	
DCB/RD ratio	0.95 ± 0.09	–		0.95 ± 0.09	–	
Length of DCB balloon (mm)	18.51 ± 4.93	–		18.84 ± 5.22	–	
Inflation pressure (bar)	8.31 ± 1.10	–		8.25 ± 1.06	–	
DCB + DES (planned)^†^	99 (39.44%)	–		69 (34.67%)	–	
SB dissection after DCB intervention (%)			-			
None	184 (73.31%)	–		141 (70.85%)	–	
Type A	39 (15.54%)	–		34 (17.09%)	–	
Type B	19 (7.57%)	–		17 (8.54%)	–	
Type C	5 (1.99%)	–		5 (2.51%)	–	
Type D	3 (1.20%)	–		1 (0.50%)	–	
Type E	1 (0.40%)	–		1 (0.50%)	–	
SB bailout stenting	9 (3.59%)	–	–	7 (3.52%)	–	–
Final strategy			–			–
Hybrid strategy (DES + DCB)	251 (100.00%)	–		199 (100.00%)		
DK crush	–	239 (30.18%)		–	113 (28.39%)	
Culotte	–	173 (21.84%)		–	90 (22.61%)	
T stent	–	33 (4.17%)		–	18 (4.52%)	
Kissing stent	–	9 (1.13%)		–	4 (1.01%)	
1-stent strategy	–	338 (42.68%)		–	173 (43.47%)	
Final kissing inflation	246 (98.01%)	721 (91.04%)	<0.001	197 (98.99%)	360 (90.45%)	<0.001
POT performed	246 (98.01%)	721 (91.04%)	<0.001	197 (98.99%)	360 (90.45%)	<0.001
Staged PCI	97 (38.65%)	314 (39.65%)	0.777	84 (42.21%)	166 (41.71%)	0.907
Complete revascularization	204 (81.27%)	639 (80.68%)	0.835	161 (80.90%)	315 (79.15%)	0.614
Procedural IVUS use	108 (43.03%)	345 (43.56%)	0.882	87 (43.72%)	173 (43.47%)	0.953
Procedural time (min)	66.49 ± 33.46	75.83 ± 30.92	<0.001	65.50 ± 32.34	77.49 ± 30.72	<0.001
Glycoprotein IIb/IIIa inhibitor used	128 (51.00%)	392 (49.49%)	0.679	104 (52.26%)	198 (49.75%)	0.563

*Plus–minus values are means ± SD. DCB, drug-coated balloon; DES, drug-eluting stent; RD, reference diameter; MV, main vessel; SB, side branch; LM, left main; DK, double kissing; POT, proximal optimization technique; PCI, percutaneous coronary intervention; IVUS, intravascular ultrasound.

^†^For diffuse lesion in SB, DCB + DES implantation was initially selected after SB lesion preparation. DCB angioplasty was done first, followed by stent deployment to ensure complete coverage of the dissection and severe elastic recoil segment (residual stenosis > 30%). The distance between the proximal end of the stent and the ostium of SB ≥ 3 mm.

Lesion preparation of the MV and SB was done for all lesions. The proportion of non-compliant balloons for SB preparation in the DCB group was significantly higher than in the DES group (82.41% *vs.* 40.45%, *P* < 0.001), which is consistent with clinical practice. More subjects underwent kissing inflation and POT in the DCB group compared with the DES group. In the DCB group, a DCB + DES strategy in SB was initially selected for 69 (34.67%) patients due to diffuse SB lesions. The bailout stenting rate was low in the DCB group (3.59%) due to appropriate lesion preparation. The rates of staged PCI and complete revascularization were similar in the 2 groups, although procedural times were longer in the DES group than in the DCB group. Furthermore, 260 (43.55%) patients underwent intravascular ultrasound (IVUS) guidance during the procedure.

### Clinical outcomes

At 30-day follow-up, TLF occurred more frequently in the DES group than in the DCB group (3.52% *vs.* 0.50%; *P* = 0.026). There were no statistical differences regarding the incidence of cardiac death, TVMI, CD-TLR, and ST (*P*s > 0.05).

**FIGURE 3:**
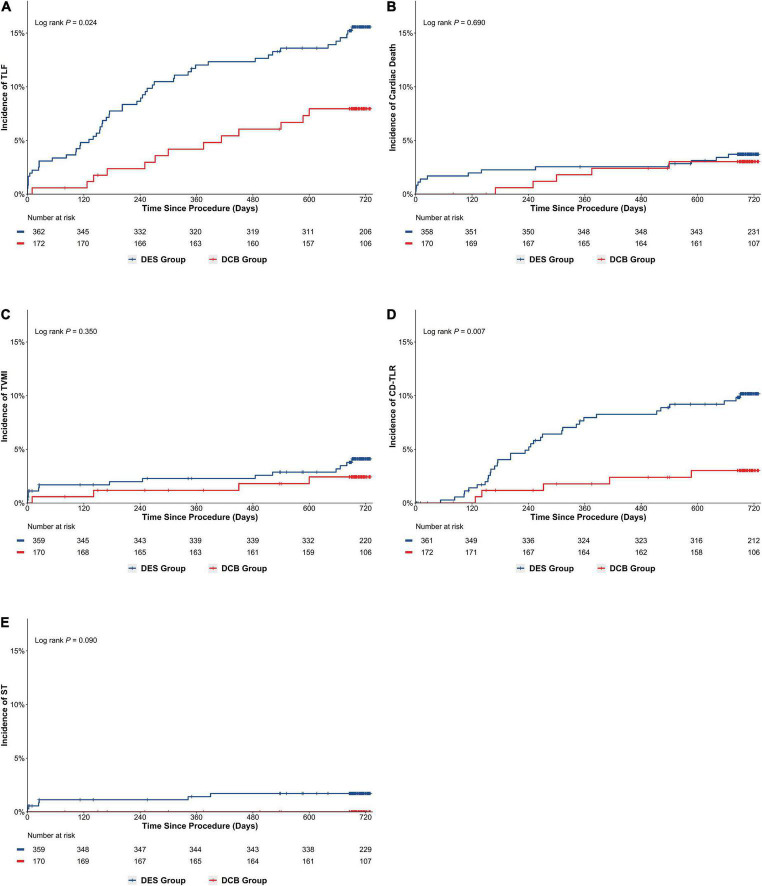
Cumulative risks of the study outcomes in the matched cohort (Kaplan–Meier time-to-first event curves). **(A)** TLF; **(B)** cardiac death; **(C)** TVMI; **(D)** CD-TLR; **(E)** ST. TLF, target vessel failure; TVMI, target vessel myocardial infraction; CD-TLR, clinically driven target lesion revascularization; ST, stent thrombosis.

**FIGURE 4:**
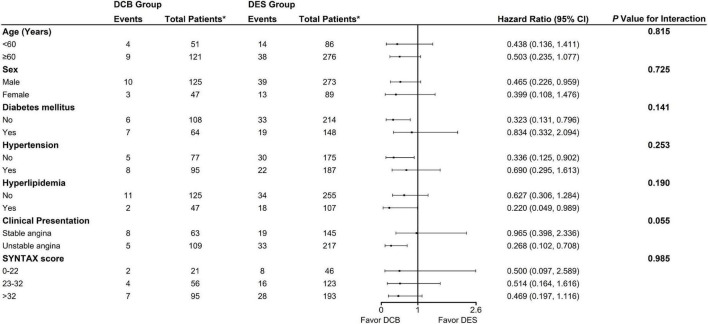
Subgroup analysis: Risk of primary outcome among propensity-score matched cohort. “*Total patients” means numbers of patients at follow up. DCB, drug-coated balloon; DES, drug-eluting stent; SYNTAX, synergy between percutaneous coronary intervention with taxus and cardiac surgery.

Following up on 89.36% of patients for a median of 729 days revealed TLF incidence rates of 8.64 and 14.64% in DCB and DES groups, respectively [Odds ratio (OR), 0.551; 95% Confidence interval (CI), 0.330–0.921; *P* = 0.021]. After PSM, the DCB group exhibited lower TLF incidence than the DES group (7.56% *vs.* 14.36%; OR, 0.487; 95% CI, 0.258–0.922; *P* = 0.025; Log-rank *P* = 0.024) (Table 4 and Figure 3A). The results were largely similar in the subgroup analyses based on selected characteristics. Moreover, there were no significant interactions between subgroups for the 2-year rate of TLF (Figure 4).

**TABLE 4:** Risk of clinical outcomes at 30-day and long-term follow-up*.

Endpoint	All patients				Propensity-matched sample			
		
	DCB group (*n* = 251)	DES group (*n* = 792)	Odds ratio (95% CI)	*P*-value	DCB group (*n* = 199)	DES group (*n* = 398)	Odds ratio (95% CI)	*P*-value
**30-day follow up, n%**	251 (100.00%)	792 (100.00%)	–	–	199 (100.00%)	398 (100.00%)	–	–
TLF^†^	1 (0.40%)	26 (3.28%)	0.118 (0.016–0.873)	0.012	1 (0.50%)	14 (3.52%)	0.139 (0.018–1.061)	0.026
Cardiac death	0 (0.00%)	9 (1.14%)	–	0.124	0 (0.00%)	6 (1.51%)	–	0.186
TVMI	1 (0.40%)	15 (1.89%)	0.207 (0.057–1.576)	0.138	1 (0.50%)	6 (1.51%)	0.330 (0.039–2.760)	0.434
Periprocedural	0 (0.00%)	10 (1.26%)	–	0.130	0 (0.00%)	4 (1.01%)	–	0.307
Non-periprocedural	1 (0.40%)	15 (0.63%)	0.630 (0.073–5.414)	1.000	1 (0.50%)	2 (0.50%)	1.000 (0.090–11.095)	1.000
Clinically driven TLR	0 (0.00%)	2 (0.25%)	–	1.000	0 (0.00%)	0 (0.00%)	–	–
Stent thrombosis	0 (0.00%)	10 (1.26%)	–	0.130	0 (0.00%)	4 (1.01%)	–	0.307
Definite	0 (0.00%)	2 (0.25%)	–	1.000	0 (0.00%)	0 (0.00%)	–	–
Probable	0 (0.00%)	8 (1.01%)	–	0.210	0 (0.00%)	4 (1.01%)	–	0.307
**1-year follow up, n%**	236 (94.02%)	762 (96.21%)	–	0.137	187 (93.97%)	385 (96.73%)	–	0.112
TLF^†^	10 (4.24%)	75 (9.82%)	0.406 (0.207–0.800)	0.007	6 (3.21%)	42 (10.91%)	0.271 (0.113–0.649)	0.002
Cardiac death	2 (0.85%)	16 (2.10%)	0.399 (0.091–1.746)	0.271	2 (1.07%)	9 (2.34%)	0.452 (0.097–2.112)	0.517
TVMI	3 (1.27%)	19 (2.49%)	0.504 (0.148–1.717)	0.264	2 (1.07%)	8 (2.08%)	0.509 (0.107–2.423)	0.511
Clinically driven TLR	7 (2.97%)	49 (6.41%)	0.446 (0.199–0.998)	0.044	3 (1.60%)	27 (7.01%)	0.216 (0.065–0.722)	0.006
Stent thrombosis	0 (0.00%)	13 (1.71%)	–	0.047	0 (0.00%)	5 (1.30%)	–	0.178
Definite	0 (0.00%)	4 (0.52%)	–	0.578	0 (0.00%)	1 (0.26%)	–	1.000
Probable	0 (0.00%)	9 (1.18%)	–	0.126	0 (0.00%)	4 (1.04%)	–	0.309
All-cause death	5 (2.12%)	21 (2.76%)	0.764 (0.285–2.048)	0.591	5 (2.67%)	10 (2.60%)	1.030 (0.347–3.058)	1.000
**2-year follow up, n%**	218 (86.85%)	714 (90.15%)	–	0.140	170 (85.43%)	358 (89.95%)	–	0.103
TLF^†^	19 (8.64%)	106 (14.64%)	0.551 (0.330–0.921)	0.021	13 (7.56%)	52 (14.36%)	0.487 (0.258–0.922)	0.025
Cardiac death	7 (3.21%)	30 (4.20%)	0.756 (0.328-1.747)	0.512	5 (2.94%)	13 (3.63%)	0.804 (0.282–2.293)	0.683
TVMI	5 (2.29%)	29 (4.05%)	0.556 (0.213–1.454)	0.225	4 (2.35%)	14 (3.90%)	0.594 (0.192–1.832)	0.359
Clinically driven TLR	9 (4.09%)	70 (9.68%)	0.398 (0.195–0.810)	0.009	5 (2.91%)	34 (9.42%)	0.288 (0.111–0.750)	0.007
Stent thrombosis	0 (0.00%)	17 (2.38%)	–	0.018	0 (0.00%)	6 (1.67%)	–	0.184
Definite	0 (0.00%)	6 (0.84%)	–	0.345	0 (0.00%)	1 (0.28%)	–	1.000
Probable	0 (0.00%)	11 (1.54%)	–	0.077	0 (0.00%)	5 (1.39%)	–	0.182
All-cause death	12 (5.50%)	41 (5.74%)	0.956 (0.493–1.854)	0.894	10 (5.88%)	19 (5.31%)	1.115 (0.507–2.453)	0.786

*DCB, drug-coated balloon; DES, drug-coated balloon; CI, confidence interval; TLF, target lesion failure; TVMI, target vessel myocardial infarction; TLR, target lesion revascularization; MI, myocardial infraction.

^†^TLF defined as the composite outcome of cardiac death, target vessel myocardial infarction, and clinical driven target lesion revascularization.

After matching, KM analysis (Table 4 and Figure 3) revealed that the cumulative rate of CD-TLR (2.91% *vs.* 9.42%; OR, 0.288; 95% CI, 0.111–0.750; *P* = 0.007; Log-rank *P* = 0.007) was significantly lower in the DCB group than in the DES group at 2 years. However, no significant difference was found between the DCB and DES groups in the incidence of cardiac death (2.94% *vs.* 3.63%; *P* = 0.683), TVMI (2.35% *vs.* 3.90%; *P* = 0.359), ST (0.00% *vs.* 1.67%; *P* = 0.184) or all-cause death (5.88% *vs.* 5.31%; *P* = 0.786).

### Quantitative coronary angiographic measurement

Angiographic follow-up was completed in 65.96% (688/1043) of patients. Results from the QCA data analysis are shown in Tables 2, 5. Baseline characteristics as assessed by QCA were comparable between the two groups. MLD post-intervention and acute lumen gain were similar in both groups, including MV and SB. During follow-up angiography, the MLD of SB in the DES group was smaller than the DCB group (2.05 ± 0.72 mm *vs.* 2.32 ± 0.71 mm, *P* < 0.001) while LLL was larger (0.42 ± 0.62 mm *vs.* 0.13 ± 0.42 mm, *P* < 0.001). Results were comparable before and after PSM.

**TABLE 5:** Quantitative coronary angiographic (QCA) results before and after propensity-score matching*.

Variable	All patients			Propensity-matched sample		
		
	DCB group (*n* = 251)	DES group (*n* = 792)	*P*-value	DCB group (*n* = 199)	DES group (*n* = 398)	*P*-value
Lesions performed angiographic follow up	176 (70.12%)	512 (64.65%)	0.111	136 (68.34%)	258 (64.82%)	0.392
**Main vessel**						
Post-intervention MLD (mm)	3.12 ± 0.22	3.14 ± 0.21	0.084	3.14 ± 0.22	3.10 ± 0.22	0.016
Acute lumen gain (mm)	2.19 ± 0.35	2.18 ± 0.37	0.318	2.20 ± 0.39	2.19 ± 0.36	0.728
Follow up MLD (mm)	2.89 ± 0.53	2.91 ± 0.48	0.606	2.85 ± 0.54	2.93 ± 0.49	0.170
Late lumen loss (mm)	0.24 ± 0.45	0.23 ± 0.42	0.817	0.25 ± 0.48	0.22 ± 0.42	0.523
**Side branch**						
Post-intervention MLD (mm)	2.50 ± 0.55	2.45 ± 0.40	0.550	2.47 ± 0.40	2.48 ± 0.52	0.708
Acute lumen gain (mm)	1.53 ± 0.58	1.46 ± 0.48	0.132	1.46 ± 0.47	1.53 ± 0.57	0.132
Follow up MLD (mm)	2.34 ± 0.71	2.04 ± 0.70	<0.001	2.32 ± 0.71	2.05 ± 0.72	<0.001
Late lumen loss (mm)	0.12 ± 0.40	0.42 ± 0.59	<0.001	0.13 ± 0.42	0.42 ± 0.62	<0.001

*Plus–minus values are means ± SD. DCB, drug-coated balloon; DES, drug-coated balloon; MLD, minimal lumen diameter.

### Sensitivity analysis

During analysis of PCI with DCB versus the 2-stent technique (225 matched pairs), the DCB group was associated with lower risks of TLF (9.60% *vs.* 17.14%, *P* = 0.026), CD-TLR (4.55% *vs.* 11.90%, *P* = 0.007) and ST (0.00% *vs.* 2.87%, *P* = 0.031) while the risks of other outcomes were similar (Supplementary Table 1). During analysis of PCI with DCB versus provisional stenting (202 matched pairs), DCB was associated with significantly lower rates of TLF (7.39 and 14.20%, respectively; *P* = 0.039), CD-TLR (3.41 and 8.57%, respectively; *P* = 0.041), and ST (0.00 and 2.89%, respectively; *P* = 0.030) but similar risks of other outcomes (Supplementary Table 2).

## Discussion

This is the first multicenter research to investigate the viability of a hybrid technique employing DES in the main vessel and DCB in the side branch for the treatment of patients with LM true bifurcation lesions, as far as we are aware. Importantly, we found that compared to the DES group, patients treated with DCB had decreased rates of TLF and CD-TLR at 2 years. The incidence rates of cardiac death, TVMI, ST, and all-cause mortality were comparable between the two groups, indicating that DCB (hybrid approach) was both safe and effective for treating *de novo* LM true bifurcation lesions. Therefore, DCB may be an alternative to stenting or perhaps the first-choice therapy for individuals who are qualified. For DCB/DES-treated SB, we discovered that the LLL at follow-up was greater in the DES group than in the DCB group. Due to the fact that our results are based on PSM, it is unlikely that they are the result of negative confounding. In addition, the validity of these data was validated using subgroup analysis and sensitivity analysis techniques.

Given the EBC’s KISSS (keep it simple, swift, and safe) recommendation, provisional SB stenting should be considered the standard therapeutic approach (19). However, the double-kissing crush approach offers the most favorable outcome data for LM true bifurcation lesions (20, 21). During clinical practice, deciding whether to execute SB dilation following crossover stenting in the LM can be challenging. In accordance with the “provisional” method, SB intervention is advised if the SB outcome is deemed sub-optimal. However, defining a “sub-optimal” result for the LCx ostium is challenging and undefined. Even in the absence of a sub-optimal SB result, the need of eliminating stent struts from the SB ostium in order to improve access to the LCx remains contentious. In fact, the presence of “floating” struts across the ostium may aid in the development of “fenestrated” LCx ostial restenosis. In contrast, a thorough registry of patients treated with crossover stenting from LM to the left anterior descending (LAD) branch indicated that the 5-year cumulative incidence of TLR was not significantly different between the kissing balloon and non-kissing balloon groups (22). Therefore, the appropriate management of coronary bifurcation lesions remains debatable, especially with LM true bifurcations (23).

Drug-coated balloon are emerging devices with established efficacy in ISR, *de novo*, and small vessels that may provide potential advantages in bifurcation PCI and circumvent the restrictions encountered with this group of lesions (24, 25). In the last several years, many clinical trials and registries have studied the efficacy of DCB in treating bifurcation lesions. In bifurcation lesions, the postulated function of DCB is to retain the simplicity of provisional stenting while reducing SB restenosis. However, it has been observed that the combination of pre-dilatation with a DCB and MV stenting with a bare metal stent is inferior to DES plus uncoated balloon due to unsatisfactory outcomes in the MV and equivalent outcomes in the side branch (11, 26). Thus, there are presently two DCB therapy options for bifurcation lesions: (1) DCBs in both MV and SB, and (2) DCB in SB and DES in MV.

Schulz et al. (27) investigated a DCB-only strategy initially, reporting a low incidence of SB and MV restenosis (3.3 and 6.6%, respectively) using second-generation DCB. Furthermore, a reduced prevalence of MACE (7.7%) was also observed, with 33% of the treated lesions located at the LM bifurcation. In the PEPCAD-BIF trial (28), after SB and MV predilatation, patients were randomly allocated to undergo either a DCB therapy of the MV and SB or plain balloon angioplasty. As potential benefits, the authors indicated the low incidence of TLR (31% *vs.* 9.4%), the increased physiological blood flow achieved, and the lack of carina shift. Bruch et al. (29) compared a DCB-only approach with a DCB + DES strategy. In the absence of thrombotic events, they identified a 9-month TLR of 4.6% and major adverse cardiovascular events (MACE) of 6.2% in patients treated with a DCB-only strategy. For specific bifurcation lesions, they found that therapy with DCB alone was both safe and effective.

Fifty patients in the DEBSIDE study (30) were treated with DES in the MV and the DANUBIO balloon (a new type of DCB) in the SB. After 12 months, the incidence of TLR was 10% in the MV and 2% in the SB. In contrast, the BIOLUX-I study (31) assessed the feasibility of provisional stenting with an everolimus-eluting stent (EES) in the MV and a paclitaxel-coated balloon in the SB. Following a 9-month follow-up, the MACE, TLR, and LLL were all low, and 11 of the 35 lesions were identified by core lab analysis to be true bifurcation lesions. The SARPEDON study (10) evaluated the effectiveness of treating the SB ostium with DCB following DES implantation in MV, followed by the kissing balloon technique. At 1-year of follow-up, the MACE rate was 19.0%, with three target vessel revascularization and two all-cause death. The rates of restenosis for MV and SB were 4 and 6%, respectively, with all SB restenosis occurring at the ostium.

The German Consensus Group initially suggested using DCB to treat bifurcation lesions (32). The administration of DCB maintains the natural morphology of the bifurcation, which is especially important in the carina area, and enables the uniform distribution of a high dose of the antiproliferative drug throughout the whole vessel surface. The use of DCB in the SB reduces the risk of incomplete coverage of the bifurcation region, scaffolding of the SB ostium, stent distortions in the MV by SB access, and overlapping and crushing of multiple metal layers and polymers with uncontrolled drug release compared to the other 2-stent techniques. The use of DCB provides a theoretical advantage over the plain balloon, with favorable vessel remodeling and plaque stabilization, as well as improved late angiographic findings, even with the simplest provisional approach.

Overall, currently available data are inconclusive and leave many concerns unsolved. The primary one is that the sequence of SB with DCB treatment remains unknown. In this research, a stent was first implanted in the MV. It should be borne in mind that DCB angioplasty of the SB first may complicate SB rewiring after MV stenting because the SB may be dissected during the DCB intervention. The SB ostium may have substantial residual stenosis and compromise the eventual therapeutic effect if the SB orifice is not dilated sufficiently after MV stenting. To prevent difficulties in rewiring the SB following MV stenting, the SB ostium may be protected by a jailed balloon or initially inflated with a small balloon. Moreover, SB rewiring via the distal struts of the stent is advised because this offers better scaffolding of the SB ostium and prevents stent distortion.

In addition, there are several difficulties, including SB lesion preparation and procedure (with or without final kissing ballooning or repeat POT). Appropriate lesion preparation improves acute gain, remodeling and prevents flow-limiting dissection. The SB ostium has greater calcium and fibrous tissue composition and a greater elastic recoil. In trying to attain the aim of ≤30% residual stenosis within 5 mm of the SB ostium, the average maximum balloon-vessel diameter ratio reached 0.93, which can significantly prolong the balloon expansion period in clinical practice. In this research, 30 (10.53%) patients were excluded owing to residual stenosis > 30% within 5 mm from the SB ostium. For bifurcation lesion preparation, non-compliant and plain semi-compliant balloons were used more often than scoring balloons since after implanting the MV stent, the scoring balloon may get trapped in the struts and cannot be withdrawn, or this type of balloon may cause damage to the DES polymer. Of these 30 patients, 25 still underwent palliative side branch DCB angioplasty (residual stenosis was 30–50%). The area of the side branch supply was not particularly large in some cases, with 30–50% residual stenosis, which was sufficient to provide blood supply to the corresponding myocardium; and balloon underexpansion was observed when lesion preparation at the side branch ostium. If SB stent implantation is undertaken on these individuals, it will surely lead to underexpansion or malposition of the SB stent. It is widely recognized that underexpansion or malposition of a stent is a significant cause of stent failure. In the remaining five cases, 2-stent was instead performed. During the 2-year follow-up, there were no clinical events among these patients.

Side branch dilation alone reduces MV stent volume and distorts MV stent symmetry; accordingly, a kissing balloon should be used to remedy stent distortion (33). The MV balloon should be inflated first, followed by simultaneous deflation of both balloons (34, 35). Importantly, the fractal geometry of bifurcations necessitates controlled modification of the 3D meshwork of the stent for optimal coronary bifurcation treatment (36). POT should be regarded as a routine procedure in bifurcation treatment since it permits reconstruction of the basic physiologic anatomy and facilitates wire interchange (37).

The mean SB lesion length in the DCB group was 15.51 (±7.07) mm, indicating that the DCB-only technique is not always practicable. After SB lesion preparation, a DCB + DES approach was initially adopted for 69 (34.67%) patients. This finding offers us sufficient confidence to perform enough pre-dilation without the misgivings of severe dissection.

Given that stent thrombosis at LM bifurcation lesions is critical, it is essential to minimize the risk of stent thrombosis to the greatest extent feasible. During the 2-year follow-up period, no thrombotic events occurred in the DCB group in this study. Although there is no statistical difference between the two groups, the value of 0% has a significant value.

Notably, some patients in the DCB group had a negative LLL of SB. Nonetheless, the fundamental cause could not be elucidated, although it is widely thought to result from vascular remodeling. Moreover, even if a severe dissection occurs during DCB angioplasty, switching to a 2-stent technique is not needed. The method of bailout stenting used in this study differs from earlier studies. It is well-established that the bailout stent should always be located at least 3 mm from the ostium of SB. Moreover, a 3–5 mm distance prevents acute occlusion of the SB in the event of severe dissection or hemorrhage. Importantly, the stent covered only the segment with dissection > type B and/or residual stenosis > 30% following DCB angioplasty, which forms the basis of our innovative technique for achieving high success rates and great clinical results without the risk of acute SB occlusion. Simultaneously, this method eliminates the metal struts covering the carina by the 2-stent technique and drastically minimizes the operation duration, procedure complexity, and learning curve.

### Study limitations

Due to the non-randomized nature of this observational research, it is susceptible to selection and ascertainment bias despite our rigorous PSM. Due to the exploratory character of this work, an “*a priori*” sample size calculation was not performed. In addition, over 90% of the patients had multivessel disease, and most had undergone complete revascularization. Moreover, the complexity of lesions (median SYNTAX score was 33 [17–42]) may increase the incidence of clinical events. Based on the KM curves of TLF, TVMI, and CD-TLR, the incidence gap between the two groups grew substantially over time. Therefore, there is reason to assume that the clinical results of the DCB group will become more robust as the length of the follow-up increases. Lastly, the DCB-only method in patients with LM true bifurcation is unquestionably interesting; nonetheless, no statistical analysis could be conducted due to the lack of available data, emphasizing the need for further studies.

## Conclusion

In patients with LM true bifurcation disease undergoing PCI, the DCB group was associated with a lower risk of prespecified clinical outcomes at 2 years. In terms of both safety and efficacy, PCI with DCB was superior to other DES-only strategies. Our findings warrant further investigation in large, randomized clinical trials with long-term follow-up to define optimal treatment strategies for this special coronary lesion set.

## Data availability statement

The original contributions presented in this study are included in the article/Supplementary material, further inquiries can be directed to the corresponding authors.

## Ethics statement

The studies involving human participants were reviewed and approved by Ethics Committee of the First Affiliated Hospital of Zhengzhou University. The patients/participants provided their written informed consent to participate in this study.

## Author contributions

CQ, WL, ZH, JD, and LP contributed to conception and design of the study. LP, WL, SP, XW, YS, MP, XQ, GS, and PZ organized the database. LP performed the statistical analysis. LP and WL wrote the first draft of the manuscript. LP, WL, and ZH wrote sections of the manuscript. All authors contributed to manuscript revision, read, and approved the submitted version.

## Footnotes

1
http://www.r-project.org

2
http://www.empowerstats.com
