# Supplementary material for: Drug-coated balloon in the treatment of coronary left main true bifurcation lesion: A patient-level propensity-matched analysis
Source: Front Cardiovasc Med. 2022 Nov 3;9:1028007. doi: 10.3389/fcvm.2022.1028007 (PMC9669294; doi:10.3389/fcvm.2022.1028007)
Supplement: Supplementary file 1 [file Data_Sheet_1.docx]

Supplementary Material

## Table 1. DCB vs. 2-stent technique: Risk of primary and secondary outcomes

## Table 2. DCB vs. provisional stenting: Risk of primary and secondary outcomes

## Figure 1. Propensity score based on spline smoothing

**Table 1. DCB vs. 2-stent technique: Risk of primary and secondary outcomes.^*^**

| Variables | No. of Patients | No. of Follow-up Patients | No. of Patients with Events | Event Rate | Odds ratio (95% CI) | *P* value |
| --- | --- | --- | --- | --- | --- | --- |
| TLF ^†^ |  |  |  |  |  |  |
| DCB | 225 | 198 | 19 | 9.60% | 0.513 (0.283, 0.929) | 0.026 |
| 2-stent technique | 225 | 210 | 36 | 17.14% | Reference |  |
| Cardiac death |  |  |  |  |  |  |
| DCB | 225 | 196 | 7 | 3.57% | 0.733 (0.274, 1.966) | 0.536 |
| 2-stent technique | 225 | 208 | 10 | 4.81% | Reference |  |
| TVMI |  |  |  |  |  |  |
| DCB | 225 | 196 | 5 | 2.55% | 0.521 (0.175, 1.552) | 0.234 |
| 2-stent technique | 225 | 209 | 10 | 4.78% | Reference |  |
| Clinically driven TLR |  |  |  |  |  |  |
| DCB | 225 | 198 | 9 | 4.55% | 0.352 (0.160, 0.775) | 0.007 |
| 2-stent technique | 225 | 210 | 25 | 11.90% | Reference |  |
| Stent thrombosis |  |  |  |  |  |  |
| DCB | 225 | 196 | 0 | 0.00% | - | 0.031 |
| 2-stent technique | 225 | 209 | 6 | 2.87% | - |  |

^*^ DCB denotes drug-coated balloon, DES drug-coated balloon, CI confidence interval, TLF target lesion failure, TVMI target vessel myocardial infarction, TLR target lesion revascularization, and MI myocardial infraction.

^†^ TLF defined as the composite outcome of cardiac death, target vessel myocardial infarction, and clinical driven target lesion revascularization.

**Table 2. DCB vs. provisional stenting: Risk of primary and secondary outcomes.^*^**

| Variables | No. of Patients | No. of Follow-up Patients | No. of Patients with Events | Event Rate | Odds ratio (95% CI) | *P* value |
| --- | --- | --- | --- | --- | --- | --- |
| TLF ^†^ |  |  |  |  |  |  |
| DCB | 202 | 176 | 13 | 7.39% | 0.482 (0.238, 0.976) | 0.039 |
| Provisional stenting | 202 | 176 | 25 | 14.20% | Reference |  |
| Cardiac death |  |  |  |  |  |  |
| DCB | 202 | 174 | 6 | 3.45% | 0.847 (0.279, 2.573) | 0.769 |
| Provisional stenting | 202 | 173 | 7 | 4.05% | Reference |  |
| TVMI |  |  |  |  |  |  |
| DCB | 202 | 174 | 4 | 2.30% | 0.485 (0.143, 1.642) | 0.236 |
| Provisional stenting | 202 | 173 | 8 | 4.62% | Reference |  |
| Clinically driven TLR |  |  |  |  |  |  |
| DCB | 202 | 176 | 6 | 3.41% | 0.376 (0.143, 0.994) | 0.041 |
| Provisional stenting | 202 | 175 | 15 | 8.57% | Reference |  |
| Stent thrombosis |  |  |  |  |  |  |
| DCB | 202 | 174 | 0 | 0.00% | - | 0.030 |
| Provisional stenting | 202 | 173 | 8 | 2.89% | - |  |

^*^ DCB denotes drug-coated balloon, DES drug-coated balloon, CI confidence interval, TLF target lesion failure, TVMI target vessel myocardial infarction, TLR target lesion revascularization, and MI myocardial infraction.

^†^ TLF defined as the composite outcome of cardiac death, target vessel myocardial infarction, and clinical driven target lesion revascularization.

**Figure 1. Propensity score based on spline smoothing. ^*^**


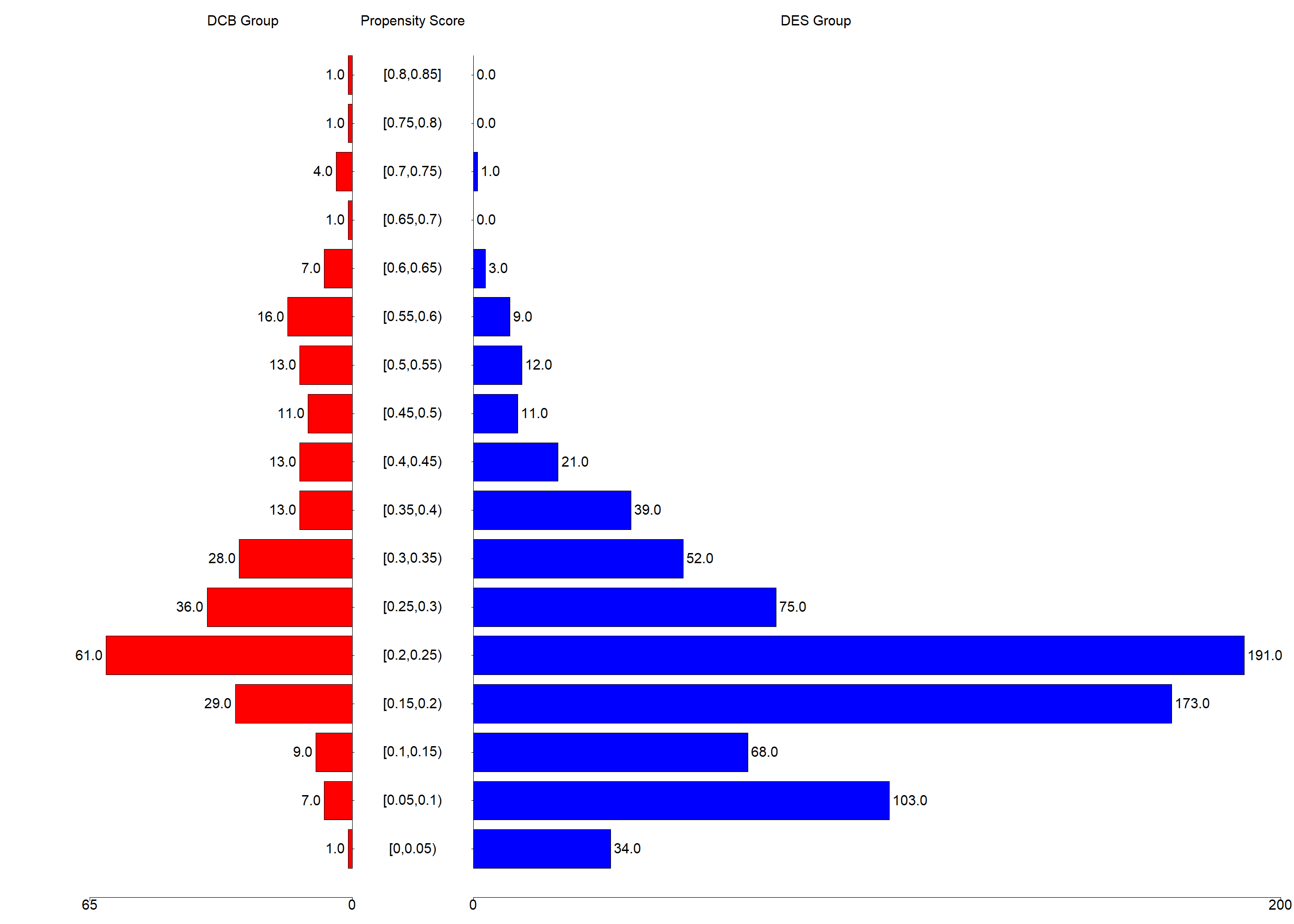


^*^ DCB denotes drug-coated balloon, DES drug-eluting stent.
